# Unraveling Delayed Puberty: A Rare Case of Congenital Hypogonadotropic Hypogonadism Masked by Celiac Disease and Plummer–Vinson Syndrome

**DOI:** 10.1002/ccr3.70952

**Published:** 2025-09-26

**Authors:** Osama Ahmad, Uzma Akbar, Fatima Sajjad, Muhammad Abdullah Ali, Umama Alam, Zaryab Bacha, Jibran Ikram, Malik W. Z. Khan

**Affiliations:** ^1^ Khyber Medical College Peshawar Pakistan; ^2^ Khyber Teaching Hospital Peshawar Pakistan; ^3^ Cleveland Clinic Foundation Cleveland Ohio USA; ^4^ Yale School of Medicine New Haven Connecticut USA

**Keywords:** congenital hypogonadotropic hypogonadism, functional hypogonadotropic hypogonadism, genetic karyotyping, Kallman syndrome

## Abstract

Congenital hypogonadotropic hypogonadism (CHH) is a rare disorder that results in delayed puberty and infertility due to impaired secretion of gonadotropin‐releasing hormone (GnRH). Here, we present a case of a 25‐year‐old male with a known history of Plummer–Vinson syndrome and celiac disease, who presented with the chief complaints of easy fatigability, shortness of breath on exertion, difficulty in swallowing, and no secondary sexual characteristics. Physical examination revealed a pale, thin, and lean male appearing significantly younger than his chronological age with notable findings of microgenitalia, absence of facial and axillary hair, decreased muscle mass, and no deepening of voice. His workup for delayed puberty, including hormonal studies, indicated hypogonadotropic hypogonadism, whereas Genetic testing confirmed a normal male karyotype. U/S scrotum showed bilateral atrophic testes. However, Neuroimaging studies revealed a normal olfactory bulb, no findings of agenesis, and hypoplasia of the olfactory sulcus. Thus, our findings in this case were consistent with and favor the diagnosis of congenital hypogonadotropic hypogonadism, also known as Idiopathic hypogonadotropic hypogonadism. It can be misdiagnosed as functional hypogonadotropic hypogonadism or simple constitutional delay of growth and puberty, especially in the presence of chronic illness. Thus, clinicians should consider CHH as one of the differential diagnoses and adopt a multidisciplinary approach when evaluating patients with delayed puberty. Timely and accurate diagnosis is crucial to initiate appropriate hormonal therapy and prevent long‐term complications such as infertility and osteoporosis.

**Summary**

- Congenital hypogonadotropic hypogonadism is a rare reproductive disorder that can be challenging to diagnose.
- This case underscores the diagnostic complexity of CHH, particularly in the presence of chronic disorders such as celiac disease and Plummer–Vinson syndrome, which can obscure the underlying etiology of delayed puberty.

## 1 Introduction

Congenital hypogonadotropic hypogonadism (CHH) is an endocrine disorder characterized by a deficiency in gonadotropin‐releasing hormone (GnRH) secretion or action, leading to impaired puberty and infertility [1]. While CHH is considered one of the most treatable forms of male infertility, it accounts for less than 1% of cases seen in fertility clinics [2]. The estimated prevalence of CHH is approximately 1 in 8000 males and 1 in 40,000 females, with both familial and sporadic cases reported [3]. CHH is classified into two main subtypes: Kallmann syndrome (KS), which is associated with anosmia or hyposmia due to defective migration of GnRH‐producing neurons, and normosmic CHH (nCHH), in which the olfactory function remains intact. The condition can be inherited in an X‐linked (11%), autosomal dominant (64%), or autosomal recessive pattern (25%), with mutations in nearly 30 different genes, such as GNRHR, ANOS1, KISS1, TAC3, and FGFR1 implicated in its pathogenesis [4, 5].

The clinical presentation of CHH varies, but hallmark features include delayed or absent puberty, micropenis and cryptorchidism in males, underdeveloped secondary sexual characteristics, infertility, and low gonadotropin and sex hormone levels. Diagnosis is established through a combination of clinical findings, hormonal assays, genetic testing, and neuroimaging, which helps differentiate CHH from other causes of hypogonadotropic hypogonadism [1].

Here, we present a case of normosmic CHH diagnosed late in a 25‐year‐old male with a known history of Plummer–Vinson syndrome (PVS) and celiac disease, who exhibited delayed puberty and hypogonadism with normal olfactory function. This case emphasizes the challenge of delayed diagnosis and treatment initiation owing to the concomitant clinical features of other comorbidities and nutritional deficiencies.

## 2 Case History

A 25‐year‐old male presented to the Khyber Teaching Hospital, Peshawar, with complaints of delayed puberty, easy fatigability, shortness of breath on exertion, and difficulty swallowing. He lacked secondary sexual characteristics, including the absence of facial and axillary hair, lack of voice deepening, and decreased muscle mass. He had been diagnosed with PVS and celiac disease several years prior and had a 10‐year history of iron‐deficiency anemia, requiring multiple blood transfusions. Despite repeated evaluations for delayed puberty in various hospitals, no definitive diagnosis had been established. He was born to nonconsanguineous parents, and he had three healthy siblings. His birth weight was approximately 3000–3500 g, and he was delivered via full‐term normal vaginal delivery. There was no significant family history of delayed puberty or endocrinological condition.

**FIGURE 1:**
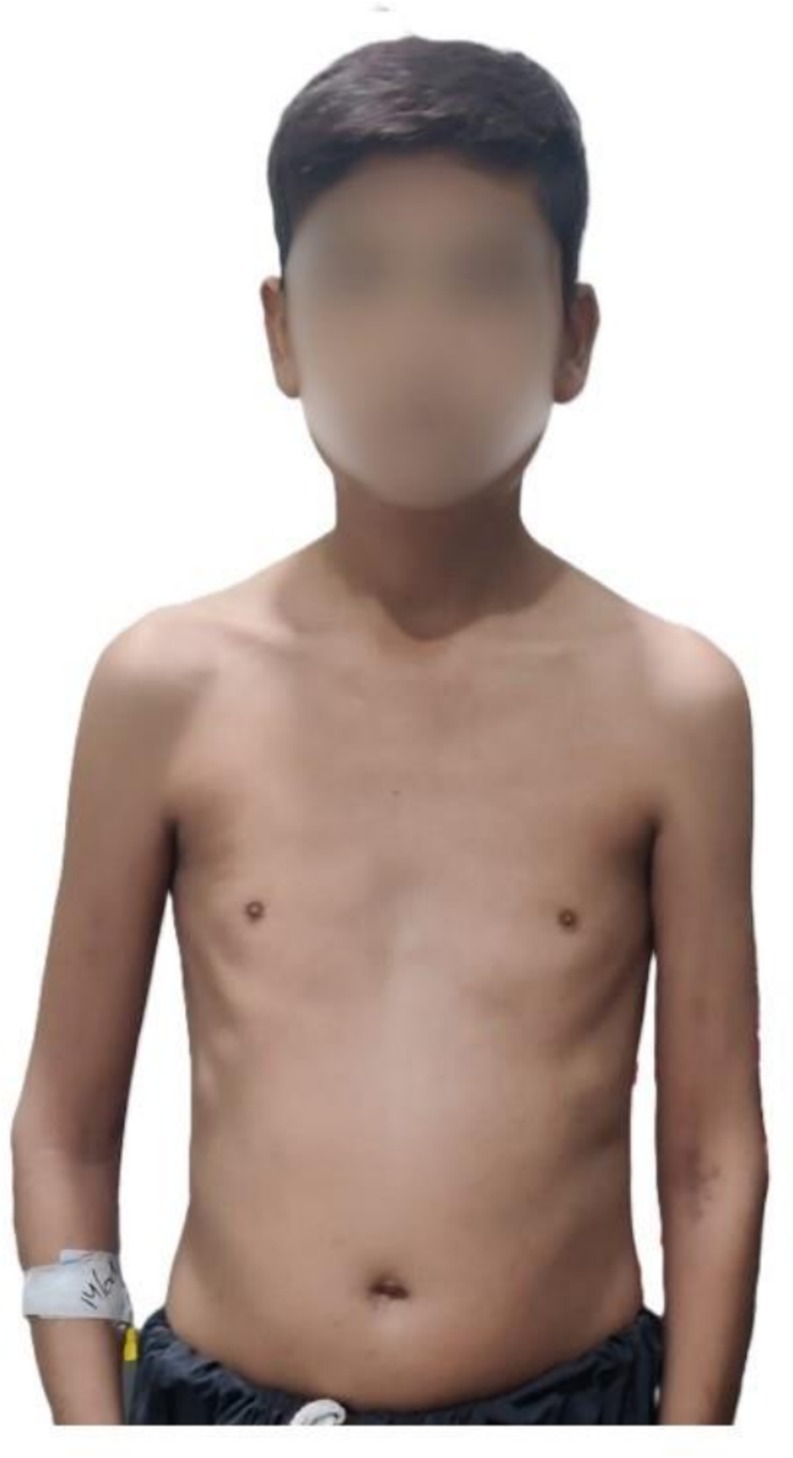
Clinical presentation of a 25‐year‐old patient with congenital hypogonadotropic hypogonadism, having a thin, lean body, decreased muscle mass, and showing no secondary sexual characteristics.

On Physical examination, his weight was 40 kg, height 160 cm, and body mass index (BMI) 15.6 kg/m^2^. His upper body segment measured 59 cm, lower body segment 81 cm, and arm span 156 cm (Figure 1). He appeared younger than his chronological age and had a lean physique with pallor and nail clubbing. Cardiovascular, respiratory, and gastrointestinal examinations were unremarkable, except for mild abdominal distention and a tender right hypochondrium. His intelligence was normal, with intact vision and a sense of smell. No facial defects, such as coloboma, cleft lip, or palate, were observed. His thyroid gland was normal in size, with no nodules or tenderness on palpation. His sexual maturation was prepubertal, with the absence of secondary sexual characteristics, such as no pubic and axillary hair, and a micropenis (4 cm length, 6 cm circumference). Both testes were present in the scrotal sac but were small, proportional to the penis size.

## 3 Investigations and Differential Diagnoses

Hormonal analysis revealed a hypogonadotropic hypogonadism profile with FSH level of 0.62 mIU/mL (normal: 1.7–12), LH < 0.10 mIU/mL (normal: 1.1–7.0), and total testosterone 0.025 ng/mL (normal: 2.5–8.5). IGF‐1 was 21.4 ng/mL (normal: 202–433), while prolactin, ACTH, TSH, and serum cortisol were within normal limits. Chromosomal analysis revealed a normal 46, XY karyotype. Laboratory investigations revealed microcytic hypochromic anemia with a hemoglobin level of 8.2 g/dL, mean corpuscular volume (MCV) of 67 fL, mean corpuscular hemoglobin (MCH) of 19.6 pg, and low serum ferritin of 7.3 μg/L with a normal retic count of 2.5%. The platelet count was 407 × 10^3^/μL, while the white blood cell count was within normal limits at 6.6 × 10^3^/μL. Renal function tests were unremarkable, with a urea level of 14.5 mg/dL and creatinine of 0.3 mg/dL. Liver function tests showed normal total bilirubin (0.17 mg/dL) and ALT (27 U/L), but a mildly elevated alkaline phosphatase level of 177 U/L. Notably, the patient had significantly low serum albumin levels at 1.12 g/dL, decreased total protein of 3.76 g/dL, and low serum calcium levels of 6.3 mg/dL, indicating poor nutritional status and possibly chronic illness.

Abdominal sonography revealed moderate gallbladder wall edema, mild splenomegaly (13 cm), moderate abdominal ascites, and bilateral pleural effusion. Echocardiography showed normal chamber sizes with no septal defects, and normal function (LVEF: 65%). Previous investigations, including esophagogastroduodenoscopy (OGD) and duodenal biopsy, had revealed an esophageal web, confirming PVS, and histological findings consistent with celiac disease, although serological markers were negative. Scrotal ultrasound revealed bilaterally atrophic testes (Right: 0.77 ml, left: 0.74 ml) with normal vascularity and epididymal morphology, without focal lesions. Initially suspected to have Prasad's syndrome, serum zinc levels were checked. Prasad's syndrome is characterized by growth retardation, hypogonadism, hepatosplenomegaly, iron deficiency anemia, and zinc deficiency. However, normal zinc levels, medical history, and clinical findings did not favor the diagnosis of Prasad's syndrome. Thus, the presence of isolated low FSH, LH, and testosterone levels, with normal function of other pituitary hormones, suggested GnRH deficiency, consistent with CHH.

**FIGURE 2:**
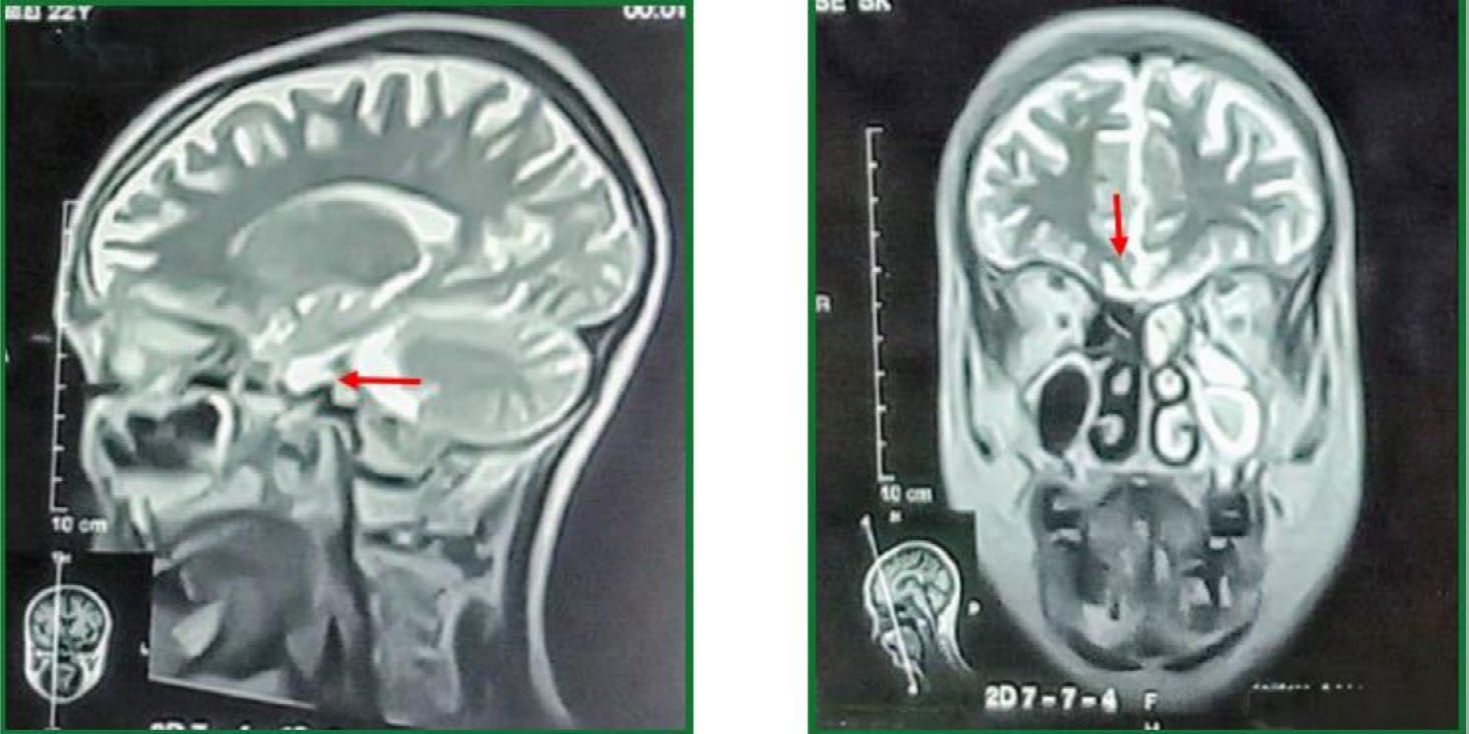
Sagittal and coronal MRI sections of the brain demonstrate a normal pituitary gland (red arrow, left) and well‐developed olfactory bulbs and olfactory sulci (red arrow, right).

Followed by MRI brain, which demonstrated a normal sella turcica, pituitary gland, optic chiasma, and suprasellar region (Figure 2). The olfactory bulbs were present and structurally normal, ruling out our suspicion of Kallmann syndrome, where MRI findings include complete agenesis of olfactory bulbs and sulci, shallow olfactory sulci, or medial orientation of the olfactory sulci. Thus, the MRI findings, patients' history and examination confirmed a diagnosis of normosmic CHH, which had remained undiagnosed despite the patient's longstanding history of delayed puberty and hypogonadism. Genetic testing for mutations commonly implicated in CHH (such as *KAL1, FGFR1*, and *GNRHR*) was not conducted due to limited resources, limiting further exploration of the etiology.

It is most challenging to distinguish between CHH and constitutional delay of puberty (CDP), particularly in early adolescence, where the clinical picture mirrors that of constitutional delay of growth and puberty. Time is a critical factor in distinguishing between these two conditions, as in CDP, spontaneous sexual maturation eventually occurs by the age of 18, whereas in CHH, sexual development is not attained without medical intervention, just like in this patient.

Another factor contributing to the late diagnosis of delayed puberty is the presence of nutritional deficiencies and chronic illnesses like Celiac disease and PVS in this case. These conditions thus lead to a misinterpretation as functional hypogonadotropic hypogonadism.

## 4 Outcome and Follow‐Up

Testosterone replacement therapy was initiated to induce secondary sexual characteristics and improve metabolic health. Initially, 2% testosterone gel was prescribed for daily topical application to the back. This was followed by intramuscular testosterone injections (250 mg every 3 weeks) for sustained androgen replacement. Anemia was managed with 200 mg IV iron sucrose for 5 days, along with multivitamin supplementation. Owing to his history of celiac disease, he had been on a gluten‐free diet for the past 5–6 years. Dietary consultation was provided to reinforce adherence and ensure adequate nutritional intake.

Regular follow‐up visits were scheduled to monitor clinical progress, adherence, and potential side effects. At the 2‐week follow‐up, the patient reported poor compliance with treatment due to financial constraints, leading to the discontinuation of therapy. Given the lifelong nature of CHH management, endocrine consultation was recommended to explore alternative treatment options, including potential gonadotropin therapy for future fertility preservation.

## 5 Discussion

CHH represents a spectrum of disorders characterized by GnRH deficiency or a rare loss of function at the receptor level, resulting in delayed puberty and infertility. Given its genetic and clinical heterogeneity, establishing a definitive diagnosis requires careful evaluation to distinguish isolated CHH from syndromic variants and to exclude secondary causes of hypogonadotropic hypogonadism [4, 6]. In this case, the presence of low gonadotropin and sex hormone levels, with otherwise intact anterior pituitary function, confirmed the diagnosis of CHH. The absence of anosmia or hyposmia, along with normal olfactory bulb morphology on MRI, ruled out KS, allowing classification under nCHH.

Although no established link has been identified among nCHH, PVS, and celiac disease, the latter can cause functional hypogonadism, adding complexity to the diagnostic process [7]. Both PVS and celiac disease contribute to chronic anemia, which can delay overall development [8]. The coexistence of celiac disease and PVS with nCHH initially shifted the clinical focus toward nutritional deficiencies and anemia, delaying the consideration of an underlying endocrinological disorder. This overlap likely contributed to the delayed recognition of CHH, underscoring the importance of a systematic approach and heightened clinical suspicion when evaluating patients with multiple systemic conditions.

Another notable finding was the absence of cryptorchidism, a feature observed in approximately 70% of males with CHH, particularly in cases diagnosed during infancy. Cryptorchidism is attributed to the crucial role of prenatal testosterone exposure in testicular descent [9]. The absence of this feature in our patient suggests a less severe prenatal androgen deficiency or a later‐onset GnRH defect [1].

Systemic illnesses, nutritional deficiencies, and genetic syndromes can suppress the hypothalamic‐pituitary‐gonadal (HPG) axis, thereby mimicking CHH [1, 4]. Chronic endocrine dysfunctions, such as hypothyroidism and hyperprolactinemia, along with conditions like uncontrolled diabetes mellitus, chronic kidney disease, and liver cirrhosis, can lead to secondary hypogonadotropic hypogonadism. Additionally, genetic syndromes such as Prader–Willi syndrome and rare variants of Klinefelter syndrome may present with hypogonadism [10]. However, the absence of characteristic dysmorphic facial features, normal intellectual ability, and normal karyotype in our patient made syndromic causes unlikely. Normal thyroid function, prolactin levels, and adrenal function excluded secondary endocrine causes, while normal MRI findings and intact pituitary hormone levels ruled out structural hypothalamic‐pituitary abnormalities (pituitary adenoma, etc.).

Distinguishing nCHH from KS and other syndromic variants (CHARGE syndrome, septo‐optic dysplasia, BBs, etc.) is essential due to differences in genetic etiology and associated features [4]. KS is frequently linked to mutations in ANOS1, FGFR1, and PROKR2, whereas nCHH is often associated with mutations in GNRHR, TAC3, and KISS1 [3]. Although genetic testing was not performed in this case due to resource limitations, the patient's phenotype was consistent with nCHH, given the isolated GnRH deficiency without additional syndromic manifestations such as craniofacial anomalies, renal agenesis, or hearing impairment.

The primary goals of CHH management are to induce puberty, maintain secondary sexual characteristics, prevent metabolic complications, and preserve fertility. Delayed treatment of CHH increases the risk of osteoporosis, dyslipidemia, insulin resistance, and visceral adiposity, emphasizing the importance of early intervention [6]. Testosterone replacement therapy (TRT) is standard for virilization and metabolic health. Gonadotropin or pulsatile GnRH therapy is required for spermatogenesis [11]. In this case, financial constraints led to the discontinuation of TRT, highlighting the need for affordable treatment options.

Selective estrogen receptor modulators (SERMs) such as clomiphene citrate may provide a cost‐effective alternative by stimulating endogenous LH and FSH secretion [12]. Concomitant diseases such as celiac disease and PVS should be addressed to minimize any further pressure on the sexual developmental delay.

## 6 Conclusion

Congenital hypogonadotropic hypogonadism is an uncommon reproductive disorder that poses diagnostic challenges, particularly in the presence of comorbid conditions like celiac disease and PVS, which may obscure recognition, especially in resource‐limited settings. It can be misinterpreted as Functional hypogonadotropic hypogonadism or just constitutional delay of growth and puberty. Also, differentiating nCHH from KS and other hypogonadotropic disorders requires thorough clinical evaluation, hormonal assessment, and imaging. Socioeconomic barriers affect treatment adherence, emphasizing the need for financial support programs and cost‐effective therapies. Early diagnosis and timely hormone replacement are crucial in preventing complications, while genetic testing, when available, can provide further insights into disease etiology and guide personalized management. Additionally, the potential association between celiac disease and PVS warrants further investigation in future cases.

## Author Contributions

**Osama Ahmad:** writing – original draft. **Uzma Akbar:** supervision. **Fatima Sajjad:** writing – review and editing. **Muhammad Abdullah Ali:** investigation. **Umama Alam:** visualization. **Zaryab Bacha:** investigation. **Jibran Ikram:** conceptualization. **Malik W. Z. Khan:** project administration.

## Consent

The authors certify that they have obtained all appropriate written patient consent forms. In the form, the patient has given his consent for his images and other clinical information to be reported in the journal. The patient understands that his name and initials will not be published, and due efforts will be made to conceal his identity.

## Conflicts of Interest

The authors declare no conflicts of interest.

## Data Availability

The data that support the findings of this study are available on request from the corresponding author. The data are not publicly available due to privacy or ethical restrictions.
